# Distinguishing between overdrive excited and suppressed ventricular beats in guinea pig ventricular myocardium

**DOI:** 10.3389/fphys.2015.00014

**Published:** 2015-02-18

**Authors:** Amara Greer-Short, Steven Poelzing

**Affiliations:** ^1^Department of Biomedical Engineering and Mechanics, Center for Heart and Regenerative Medicine, Virginia Polytechnic Institute and State University, Virginia Tech Carilion Research InstituteRoanoke, VA, USA; ^2^School of Biomedical Engineering and Sciences, Virginia Polytechnic Institute and State UniversityBlacksburg, VA, USA

**Keywords:** automaticity, triggered activity, overdrive excitation, overdrive suppression, hypothermia, digoxin, hypercalcemia

## Abstract

Rapid ventricular pacing rates induces two types of beats following pacing cessation: recovery cycle length (RCL) prolongation (overdrive suppression) and RCL shortening (overdrive excitation). The goals of this study were to compare common experimental protocols for studying triggered activity in whole-heart preparations and differentiate between recovery beats using a new methodology. Post-pacing recovery beat cycle length (RCL) and QRS were normalized to pre-paced R-R and QRS intervals and analyzed using a K-means clustering algorithm. Control hearts only produced suppressed beats: RCL ratio increased with rapid pacing (25 ± 4.0%, *n* = 10) without changing QRS duration. Rapid pacing during hypercalcemia + hypothermia (5.5 mM and 34°C) produced significantly earlier excited beats (53 ± 14%, *n* = 5) with wider QRS durations (58 ± 6.3%, *n* = 5) than suppressed beats. Digoxin + hypothermia (0.75 μM) produced the most excited beats with significantly earlier RCL (44 ± 3.2%, *n* = 6) and wider QRS (60 ± 3.1%, *n* = 6) ratios relative to suppressed beats. Increasing pacing further shortened RCL (30 ± 7.8%, *n* = 6). In a prospective study, TTX (100 nM) increased RCL ratio (15 ± 6.0%, *n* = 10) without changing the QRS duration of excited beats. The algorithm was compared to a cross-correlation analysis with 93% sensitivity and 94% specificity. This ECG based algorithm distinguishes between triggered and automatic activity.

## Introduction

The heart electrically excites and contracts in a specific order, as automaticity *in sino*-atrial nodal cells sets a rhythm faster than other cardiac cells. However, at times electrical activity can originate prematurely at ectopic sites, leading to arrhythmias. Early work in canine purkinje fibers using cardiac glycosides found premature low amplitude membrane depolarizations following rapid pacing (Ferrier et al., 1973; Hashimoto and Moe, 1973; Rosen et al., 1973). These small depolarizations grew in amplitude as the pacing rate was increased, and the interval between the last paced beat and the onset of the oscillation decreased (Ferrier et al., 1973; Hashimoto and Moe, 1973). Close observation led to the discovery that the onset of these small depolarizations in Purkinje fibers were concordant with the onset of ventricular arrhythmias (Rosen et al., 1973). This suggested that small depolarizations may be responsible for cardiac glycoside-induced ventricular arrhythmias. Indeed, these depolarizations, later known as delayed afterdepolarizations (DADs), could initiate an action potential if the amplitude was large enough (Ferrier et al., 1973). These action potentials initiated by DADs later became known as triggered activity (TA), as their initiation was dependent on rapid pacing or “trigger” (Cranefield, 1977). Finally, the recovery beat cycle length of TA decreases as pacing rate increases, and this is referred to as overdrive excited (OE) behavior. In contrast, rapid pacing can also overdrive suppress (OS) the recovery beat by temporarily suppressing normal automaticity (Malfatto et al., 1988; Iinuma et al., 1989), presumably by hyperpolarizing the membrane (Vassalle, 1970). Therefore, rapid pacing can produce mechanistically different recovery beats: normal (OS) and arrhythmogenic (OE).

Later observations in intact heart models discovered that glycosides decreased recovery beat cycle length following rapid pacing (Malfatto et al., 1988; Vos et al., 1990), suggesting that triggered recovery beats in intact models with glycoside toxicity were due to TA, as their behavior was consistent with data from Purkinje fibers. More generally speaking, cardiac glycosides and many other interventions such as hypercalcemia (Plummer et al., 2011), catecholamine perfusion (Priori and Corr, 1990), and myocardial infarction (Lazzara et al., 1973) are associated with an increased incidence of DADs and TA.

Importantly, the recovery beat cycle length may be insufficient to distinguish between OS and OE beats. For example, an automatic beat could occur before a triggered rhythm at slow pacing rates and be classified as a triggered rhythm. Therefore, additional criteria are necessary to distinguish between mechanistically different recovery beats. The goals of this study were to (1) Differentiate between OS and OE beats with a new methodology, (2) Develop a robust protocol for creating OE beats that can be studied, and (3) Demonstrate that OE beats can be pharmacologically manipulated.

## Materials and methods

The investigation conforms to the *Guide for the Care and Use of Laboratory Animals* published by the US National Institutes of Health (NIH Publication No. 85-23, revised 1996) and has been approved by Institutional Animal Care and Use Committee (IACUC) at Virginia Polytechnic Institute and State University.

### Guinea pig langendorff preparations

Retired breeder male guinea pigs (*n* = 37) were anesthetized with sodium pentobarbital (325 mg/kg) and injected intraperitoneally with heparin to prevent blood clotting. Atria were excised to remove competitive stimulation from atria, and ventricles were perfused in a Langendorff system with oxygenated Tyrode solution (in mM, CaCl_2_ 1.25, NaCl 140, KCl 4.56, dextrose 5.5, MgCl_2_ 0.7, HEPES 10; pH to 7.4 with approximately 5.5 mL of NaOH) at 37°C and 50 mmHg. Motion was reduced using 7.5 mM 2,3-diacetylmonoxime.

### Electrocardiography (ECG) and interventions

A volume-conducted bath electrocardiogram (ECG) similar to lead I was continuously recorded to determine recovery cycle length (RCL) and QRS duration of the QRS complex immediately after the cessation of pacing. Hearts were paced with plunge bipolar electrodes fixed in the basal interventricular septum at pacing rates from 200 to 375 bpm for 15 s. Hearts were also perfused with the glycoside digoxin (0.75 μM), high extracellular calcium (5.5 mM CaCl_2_), or the sodium channel inhibitor tetrodotoxin (TTX, 100 nM). Hearts were maintained at normal (37°C) and hypothermic (34°C) temperatures. ECG measurements were made 10 min after the start of perfusion. RCL and QRS ratios were then normalized to the pre-paced beat's cycle length (BCL) and the QRS, called RCL and QRS ratios, respectively. The recovery window was 1500 ms. A k-means two cluster analysis, which is an automated clustering algorithm that calculates the distance from each data point to a continuously calculated centroid of each cluster, was used to separate the control, hypercalcemic, digoxin, and hypothermic data into two clusters (Cluster 1 and Cluster 2).

A prospective analysis was performed for the digoxin + tetrodotoxin data set, where the centroids of Clusters 1 and 2 from the original data set (control, hypercalcemia, digoxin, hypothermia) were calculated and recovery beats for digoxin + tetrodotoxin were stratified into two clusters depending on the centroid they were closest to.

To determine sensitivity and specificity of our new algorithm, cluster classification was compared to a previously validated cross-correlation analysis as the gold-standard (Kyle et al., 2009). The maximum cross-correlation coefficient was used in this study to analyze QRS morphology similarities between Cluster 1 and 2 recovery beats and their prospective pre-paced beats. True positive was defined as OE beats with a coefficient < 0.9, false positive as OE beats with a coefficient ≥ 0.9, true negative as OS beats with a coefficient ≥ 0.9, false negative as OS beats with a coefficient < 0.9.

### Statistical analysis

Where appropriate, unpaired and paired two-tailed Student's *t*-tests with equal/unequal variance or the nonparametric Wilcoxon rank-sum test were used to analyze data significance. The *F*-test for equality of two variances was used to analyze variance significance. The Sidak correction for multiple comparisons was used. A *p* < 0.05 was considered significant. Values are reported as mean ± standard error. Retrospective power analyses were performed to determine the recommended sample sizes. The minimum number of samples per intervention was calculated using a power analyses for a 1 sided, two independent sample normal distribution test with alpha 0.05 and a power of 0.8. Minimum number of beats:

- Hypercalcemia: 5 OS and 5 OE beats
- Hypothermia + hypercalcemia: 5 OS and 5 OE beats
- Digoxin: 4 OS and 4 OE beats
- Hypothermia + digoxin: 5 OS and 5 OE beats
- Digoxin + TTX: 4 OS and 4 OE beats

## Results

### Overdrive suppression and excitation

**Figure 1:**
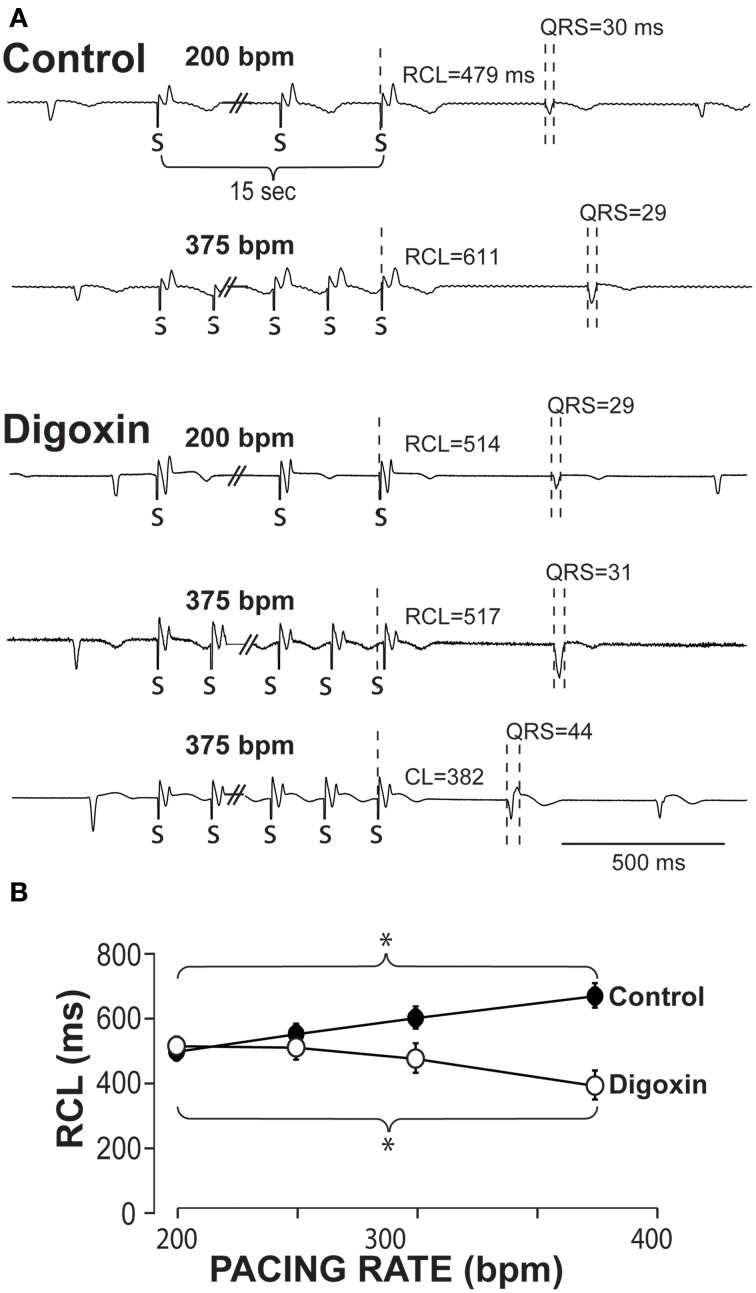
**Recovery beats characteristics in response to slow and rapid pacing. (A)** Representative volume-conducted electrocardiograms of control (top) and digoxin (bottom) at pacing rates of 200 and 375 bpm. Paced beats are marked by “S.” **(B)** Summary data of the recovery beat cycle length following different pacing rates. Control data had an increase in RCL (^*^p < 0.05, *n* = 11) as pacing rate was increased, while digoxin had a decrease in RCL (^*^p < 0.05, *n* = 12).

Example ECGs from control hearts in Figure 1A demonstrate that RCL increases when pacing rate is increased from 200 to 375 bpm. Representative ECGs in Figure 1A also demonstrate in hearts perfused with 0.75 μ M digoxin that increasing pacing can decrease the recovery beat's RCL. For all experiments, increasing pacing rate in control hearts from 200 to 375 bpm significantly increases RCL, whereas during digoxin, rapid pacing decreases RCL (Figure 1B).

**Figure 2:**
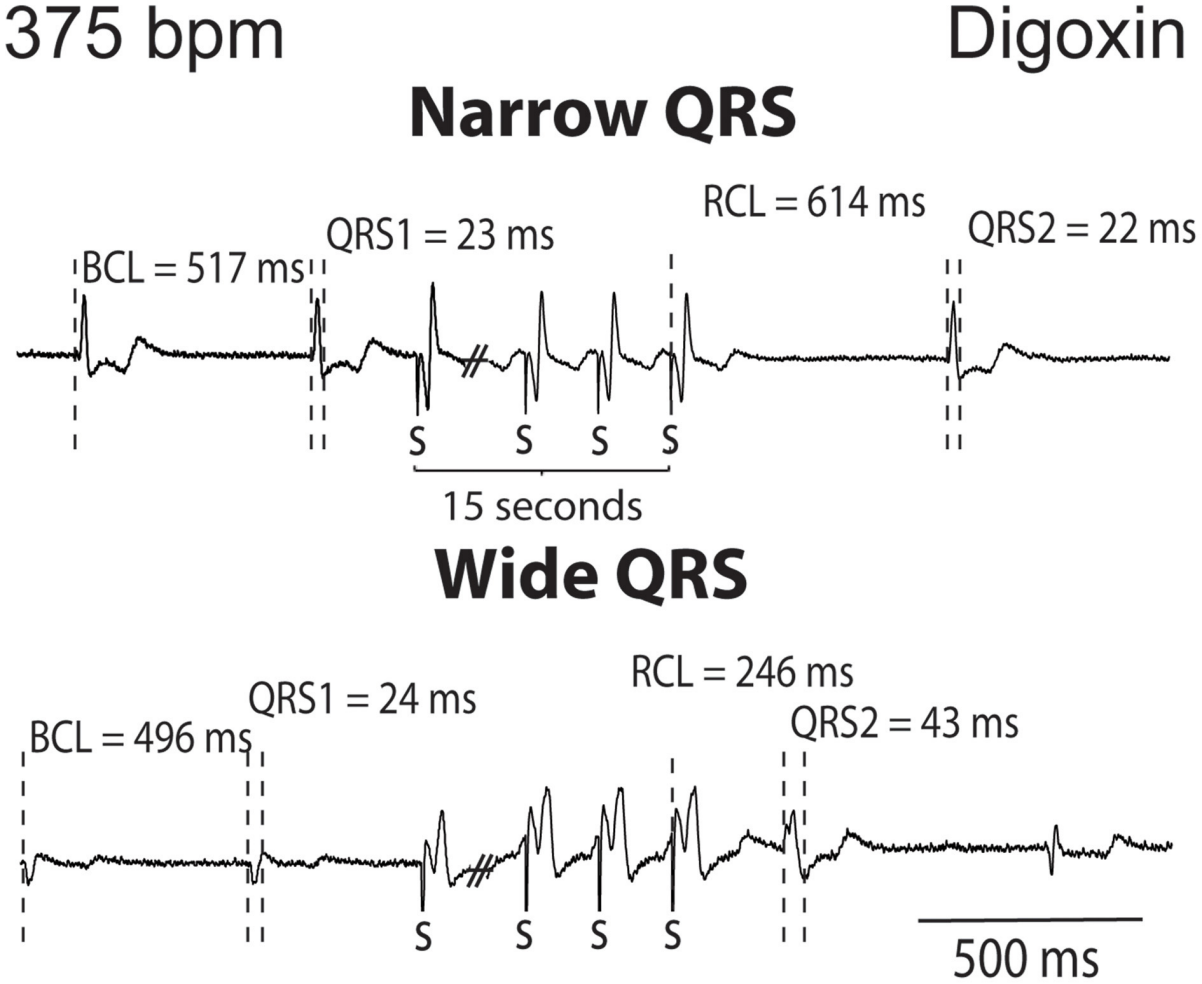
**Digoxin recovery beats can manifest a late recovery-narrow QRS duration or an early recovery-wide QRS duration**. Representative ECGs from digoxin experiments. Recovery beats with narrow and wide QRS complexes are shown in response to 15 s of rapid pacing. Figure illustrates representative cycle lengths (CL) and QRS of pre-paced beats, and the cycle length (RCL) and QRS or the recovery beat.

The QRS complexes of the recovery beat in the control hearts in Figure 1A appear similar, while the digoxin recovery beats at 375 bpm exhibit variable ECG characteristics. Specifically during rapid pacing, digoxin recovery beats can be associated with 1. narrow QRS durations and increased RCL (Figure 2, top panel) and with 2 wide QRS durations and decreased RCL (bottom panel) relative to the pre-paced beat. In summary, at 200 bpm, the RCL of digoxin recovery beats was 511 ± 94 ms and the QRS 27 ± 8 ms. At 375 bpm, RCL significantly decreased to 388 ms but the standard deviation significantly increased to 154 ms. Conversely, QRS increased to 36 ms without significantly altering standard deviation. This suggests that recovery beats in the digoxin group (Figure 1B) may be composed of beats that could be either automatic or triggered activity (TA). Therefore, it is important to stratify beat type in order to study mechanistic behaviors.

**Figure 3:**
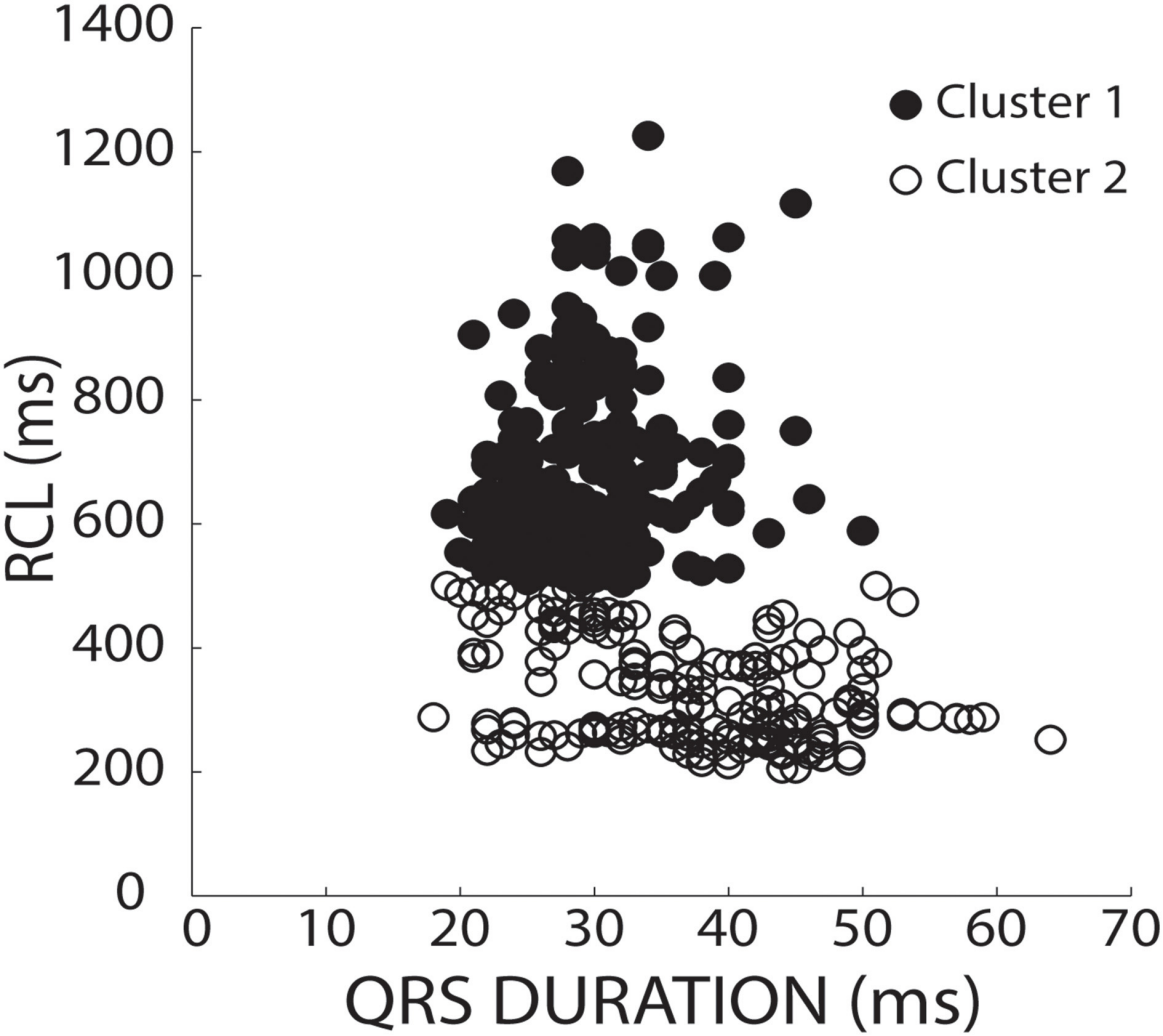
**K-means separates clusters of raw values by RCL alone**. RCL and QRS absolute values from all recovery beats are shown under both normal (37°C) and hypothermic (34°C) conditions from control, hypercalcemic, and digoxin conditions for both slow (200 bpm) and fast (375 bpm) pacing rates. Data was stratified into two clusters using a k-means clustering algorithm. As shown, the data preferentially separated in the RCL direction.

To stratify recovery beats, we used a k-means 2-cluster analysis on raw RCL and QRS values. Figure 3 demonstrates preferential clustering based on RCL values, because the range of RCL values (205 to 1226 ms) is significantly larger than the QRS duration range (18 to 64 ms). More specifically, k-means is based on each data point's distance from a centroid. Therefore the larger range has a larger effect on clustering, and as a result, clustering raw RCL and QRS reduces the influence of QRS duration.

Furthermore, pharmacologic intervention and inter-animal variability can underlie differences in the basic cycle length (BCL) and QRS morphology of native beats, and therefore the recovery beat. For example, any intervention which increased native rhythm rates would likely decrease all recovery beat RCL. This analysis could classify the recovery beat as an OE beat without accounting for the fact that BCL changed. A similar argument can be made for QRS.

**Figure 4:**
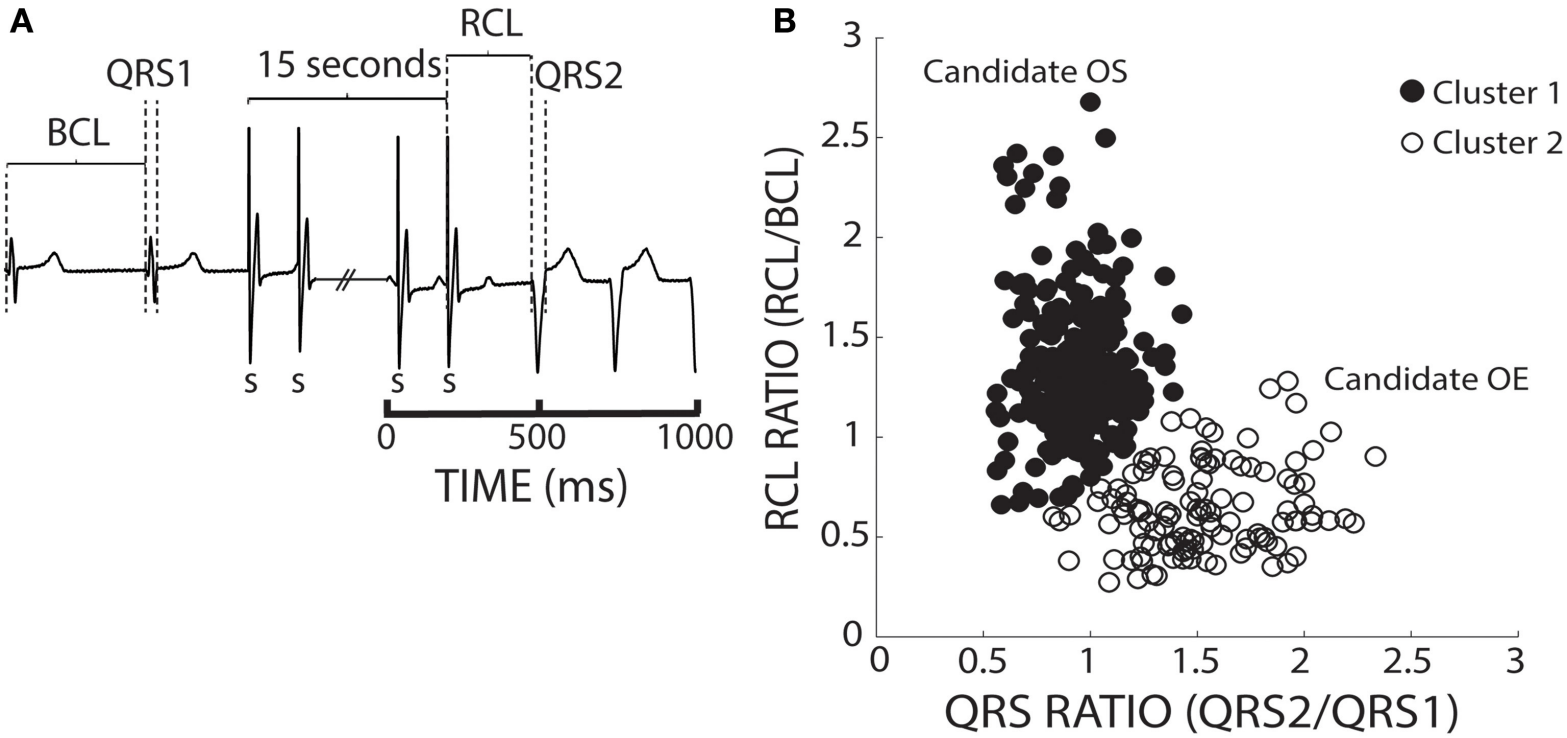
**K-means separates ratio values by both parameters. (A)** Representative ECGs showing how RCL and QRS ratios were quantified. The recovery beat's cycle length (RCL) and QRS (QRS2) were normalized to the R-R interval or basic cycle length (BCL) and QRS (QRS1). **(B)** Shown are the RCL and QRS ratios of all beats obtained from control, hypercalcemic, and digoxin interventions under both normal (37°C) and hypothermic (34°C) conditions for both slow (200 bpm) and fast (375 bpm) pacing rates. A k-means clustering algorithm was used to separate the data into 2 clusters.

To account for BCL and QRS variability, we chose to normalize recovery beat RCL and QRS. In Figure 4A, an ECG before, during, and after pacing illustrates how the BCL and QRS durations were measured prior to pacing (QRS1), and how the RCL and QRS durations were measured post-pacing (QRS2). We normalized the RCL and QRS duration of the recovery beat to the pre-paced beat's BCL and QRS duration, respectively (Figure 4A). The RCL and QRS ratios of all recovery beats from multiple interventions were then plotted and the aforementioned k-means 2-cluster analysis performed. Figure 4B demonstrates recovery beat classification, where Cluster 1 (filled circles) is composed predominantly of beats with late relative RCL (≤1) and narrow QRS complexes (~1), and Cluster 2 (open circles) is composed predominantly of beats with early RCL (=1) and wide QRS complexes (>1). The averages and standard deviations for these clusters are as follows: Cluster 1 RCL 1.31 ± 0.33 and QRS 0.94 ± 0.16 and Cluster 2 RCL 0.64 ± 0.22 and QRS 1.53 ± 0.32. These Cluster 1 beats, therefore, may be overdrive suppressed, while Cluster 2 beats may be overdrive excited.

### Control

**Figure 5:**
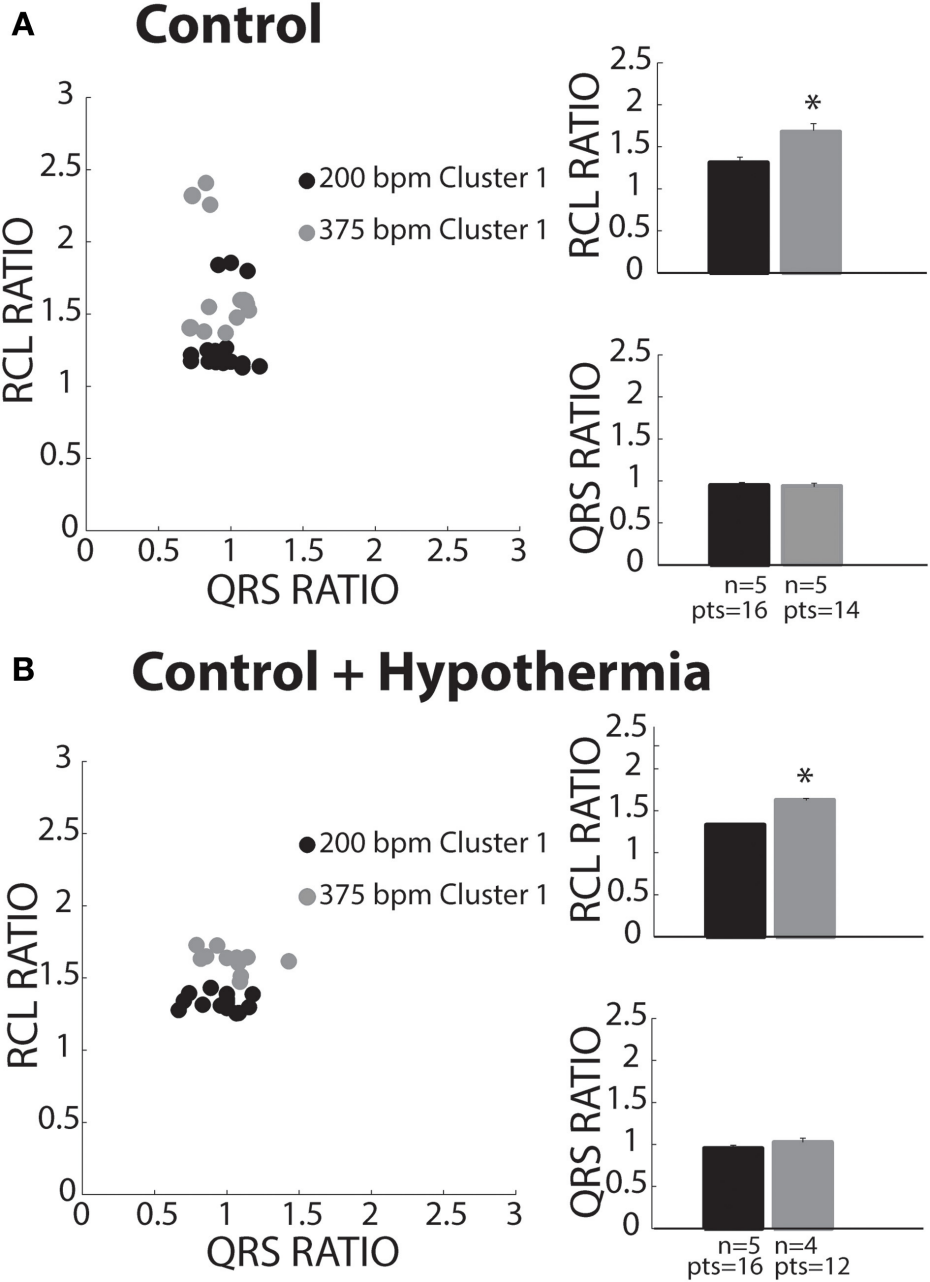
**Control recovery beats classified as OS. (A)** All slow (black circles) and fast (gray circles) paced recovery beats fell within Cluster 1 (filled circles). RCL ratio summary data of both slow (*n* = 5) and fast (*n* = 5) paced recovery beat groups. Rapid pacing rates increased the RCL ratio (^*^p < 0.05), while QRS ratio remained unchanged. **(B)** All slow (black circles) and fast (gray circles) paced recovery beats fell within Cluster 1. Summary data of RCL ratio from recovery beats shows that rapidly pacing (*n* = 4) increased RCL ratio relative to slow pacing rates (^*^p < 0.05, *n* = 5), while QRS ratio did not change.

Keeping the cluster assignment for each recovery beat, we observed that control conditions only produced beats that were within Cluster 1 (Figure 5A, solid circles). Consistent with the theory of preferential overdrive suppression (OS) in normal hearts, faster pacing rates (gray circles) prolonged RCL relative to slower rates (black circles). For all experiments, rapid pacing at 375 bpm significantly increased the RCL ratio without affecting QRS duration. Furthermore, mean native and recovery QRS durations from control hearts was 29 ± 4 ms, which is comparable to *in vivo* and *ex vivo* guinea pig measurements with atria intact (Stark et al., 1993; Batey et al., 1997). Therefore, these data suggest that recovery beats in these experiments with atria removed were all OS, and the pacemaker site for OS beats may be localized to the atrioventricular node or His bundles.

### Control + hypothermia

Some studies have investigated spontaneous calcium release dynamics under hypothermic conditions (Wasserstrom et al., 2010; Plummer et al., 2011), which may impact TA characteristics. Therefore, we assessed recovery beats under physiologic hypothermia (34°C). Consistent with control conditions, hypothermia only produced beats assigned to Cluster 1 (Figure 5B). Furthermore, RCL ratio was increased following faster relative to slower pacing (right panel, gray and black bars), while QRS ratio remained unchanged. As in control hearts, this is consistent with OS behavior. Lastly, hypothermia did not affect RCL or QRS ratios relative to control at normothermic conditions.

### Hypercalcemia

**Figure 6:**
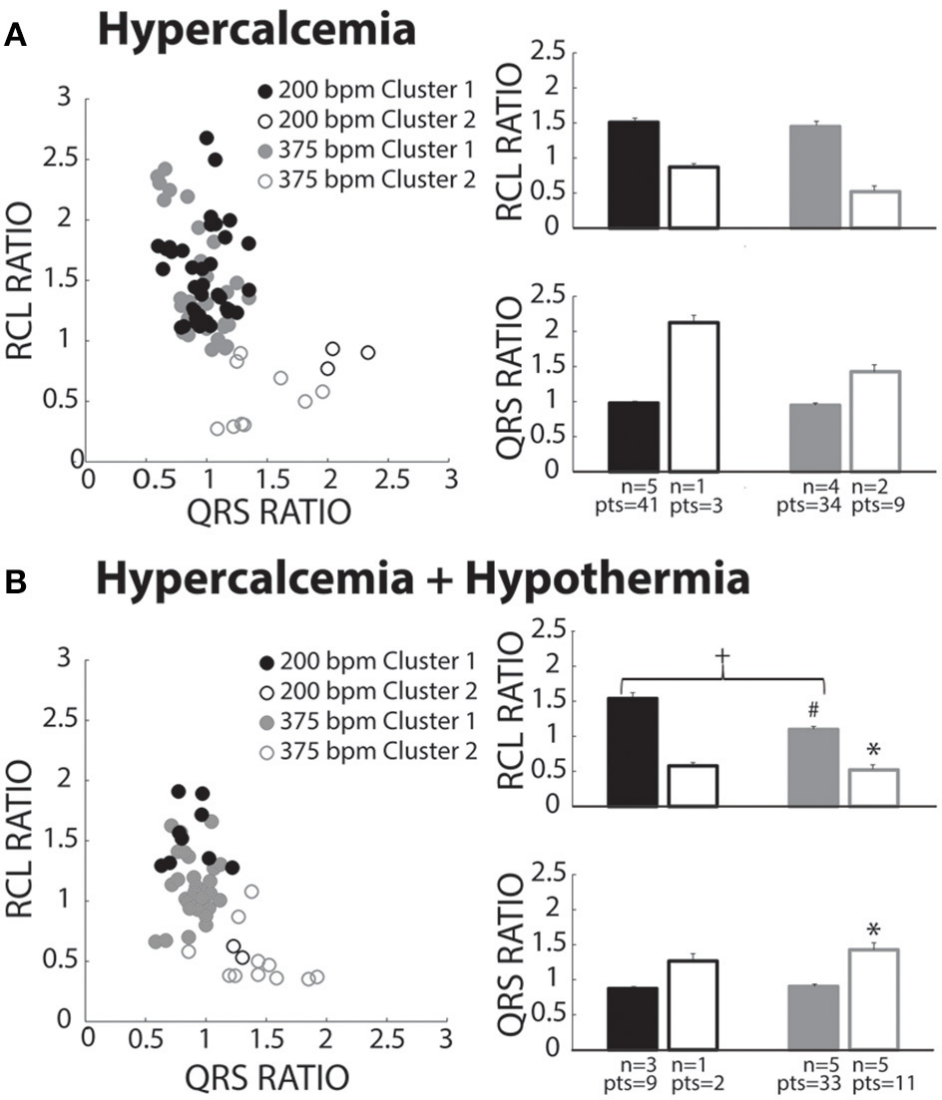
**Hypercalcemia recovery beats classified as both OS and OE**. **(A)** Both slow (black circles) and fast (gray circles) paced beats fell within both Clusters 1 (closed circles) and 2 (open circles). Beats within Cluster 2 for both the slow (*n* = 1) and fast (*n* = 2) paced groups were insufficiently powered for statistical analysis. For Cluster 1 beats, pacing rate did not significantly change RCL or QRS ratio. **(B)** Pacing at slow (black circles) and fast (gray circles) rates created beats classified in Cluster 1 (filled circles) and Cluster 2 (open circles). Statistical analyses could not be performed with the fast paced Cluster 1 group due to lack of power (*n* = 1). Rapidly paced Cluster 2 group (*n* = 5) had a smaller RCL ratio when compared to the rapidly paced Cluster 1 group (*n* = 5, ^*^p < 0.05). For Cluster 1, rapid pacing (*n* = 5) decreased the RCL ratio and increased QRS ratio relative to the slow paced group (*n* = 3, +p < 0.05). Hypercalcemia and hypothermia (*n* = 5) for the rapidly paced Cluster 1 group (solid gray bar) decreased the RCL ratio when compared to normal hypercalcemia (Figure 6A, *n* = 5, #*p* < 0.05).

Hypercalcemia has previously been shown to induce spontaneous calcium releases and TA (Wasserstrom et al., 2010; Plummer et al., 2011). Hypercalcemia (5.5 mM) under normal physiological temperature was investigated, and surprisingly, produced beats preferentially assigned to Cluster 1 (Figure 6A, closed circles). Within Cluster 1, RCL and QRS ratios were unaffected by pacing rate. Due to lack of power, Cluster 2 data is presented but not statistically compared to Cluster 1.

### Hypercalcemia + hypothermia

**Figure 7:**
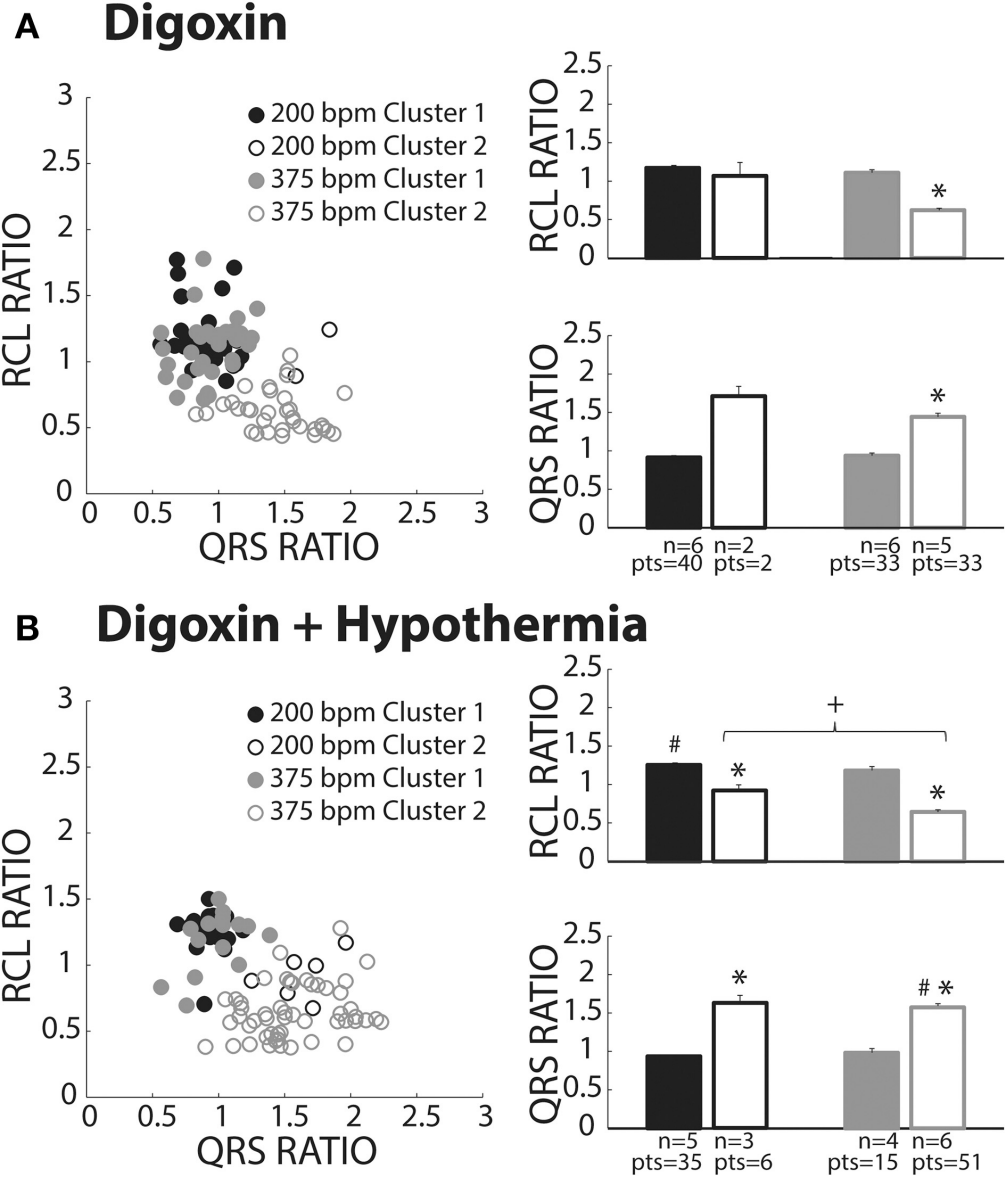
**Digoxin recovery beats classified as both OS and OE**. **(A)** Recovery beats the result of slow (black circles) and fast (gray circles) pacing rates fell within both Clusters 1 (closed circles) and 2 (open circles). Statistical analyses could not be performed with the fast paced Cluster 1 group due to lack of power (*n* = 2). RCL ratio for the rapidly paced recovery beat group in Cluster 2 (*n* = 5) was shortened when compared to Cluster 1 (*n* = 6, ^*^p < 0.05). Rapidly paced recovery beats from Cluster 2 had a larger QRS ratio relative to the Cluster 1 group (^*^p < 0.05). **(B)** Slow (black circles) and fast (gray circles) paced recovery beats fell within both Cluster 1 (filled circles) and Cluster 2 (open circles). Recovery beats the result of slow pacing in Cluster 2 (*n* = 3) had a smaller RCL ratio relative Cluster 1 (*n* = 5, ^*^p < 0.05). The same occurred for rapidly paced Cluster 2 (*n* = 6) beats relative to Cluster 1 (*n* = 4, ^*^p < 0.05). Rapidly pacing the Cluster 2 group (*n* = 6) decreased the RCL ratio relative to the slow paced Cluster 2 group (*n* = 3, +p < 0.05). Hypothermia also increased the RCL ratio for the slow paced Cluster 1 group (*n* = 5) relative to normal digoxin slow paced Cluster 1 group (Figure 7A, *n* = 6, #*p* < 0.05). QRS ratio for the slow paced Cluster 2 group increased relative to Cluster 1 (^*^p < 0.05). The same occurred for the fast paced Cluster 2 group relative to Cluster 1 (^*^p < 0.05). Hypothermia increased the QRS ratio for the rapidly paced Cluster 2 group (*n* = 6) relative to normothermia (**A**, *n* = 5, #*p* < 0.05).

Slower pacing in hypercalcemic + hypothermic hearts resulted in more recovery beats classified in Cluster 1 (Figure 6B, black filled circles). During rapid pacing, more hearts exhibited Cluster 2 beats (gray open circles), and the RCL ratio of Cluster 2 beats was significantly shorter and QRS ratio significantly wider than Cluster 1 beats. Thus, overdrive excited (OE) beats were initiated under hypercalcemia during hypothermia. Surprisingly, the RCL ratio of Cluster 1 beats was significantly shorter at faster relative to slower pacing rates (right panel, closed bars). Hypothermia significantly decreased RCL ratio for fast paced Cluster 1 beats relative to normothermia in Figure 7A (#).

### Digoxin

Digoxin produced beats classified in Clusters 1 (closed circles) and 2 (open circles, Figure 7A). Like hypercalcemia, faster pacing did not increase RCL ratio for Cluster 1 beats as evidenced by black and gray filled circle overlap in the left panel and no difference between the average RCL ratios.

At faster pacing rates (Figure 7A, gray symbols), RCL ratio was significantly shorter for Cluster 2 relative to Cluster 1 and this was paralleled by a significant increase in the QRS ratio. Cluster 2, therefore, behaved as expected of OE recovery beats.

### Digoxin + hypothermia

Digoxin during hypothermia produced beats within Clusters 1 and 2. The RCL ratio for Cluster 1 beats was not significantly affected by rapid pacing, suggesting that Cluster 1 beats were insensitive to overdrive pacing (Figure 7B, filled black and gray bars). This is similar to observations in normothermic digoxin conditions.

Regardless of pacing rate, Cluster 2 beats (Figure 7B, open bars) manifested decreased RCL and increased QRS ratios relative to the Cluster 1 beats (filled bars). Importantly, increasing pacing rate shortened the RCL ratio of Cluster 2 beats, as evidenced by the comparison of open bars (+, black and gray) without changing QRS ratio. Thus, Cluster 2 beats behaved as expected of OE beats.

With respect to normothermic digoxin in Figure 7A, hypothermic digoxin increased RCL ratio for Cluster 1 beats following relatively slower pacing (Figure 7B, #). Additionally, hypothermia increased the QRS ratio of Cluster 2 beats relative to normothermic digoxin (#).

### Overdrive excitation incidence

**Figure 8:**
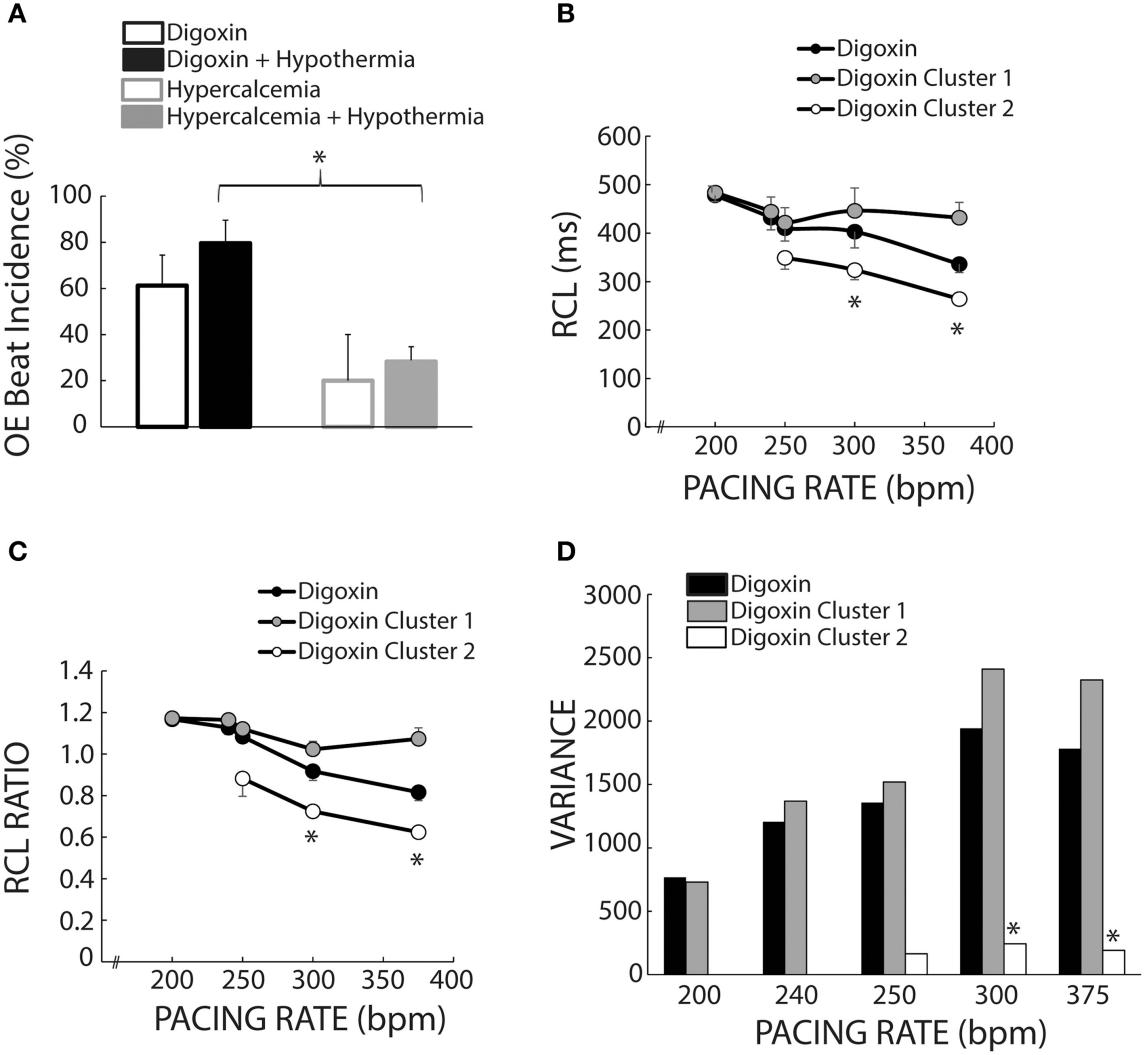
**Cluster separation reveals distinct OS and OE behaviors during Digoxin. (A)** Average OE beat occurrence. Hypothermia did not significantly alter OE beat occurrence under either digoxin or hypercalcemic conditions. OE beat for hypothermic digoxin (*n* = 5) were significantly more frequent when compared to hypothermic hypercalcemia (*n* = 5, ^*^p < 0.05). **(B)** Recovery beat cycle lengths before stratification in black circles. After stratification there are significant difference between Clusters 1 (gray circles) and 2 (white circles) at 300 and 375 bpm. Of note, only data with at least *n* = 3 are plotted in panels **(B–D)**. **(C)** RCL ratios for the entire dataset (black circles), Cluster 1 (gray circles), and Cluster 2 (white circles). Cluster 2 RCL ratios were significantly smaller when compared to Cluster 1 at 300 and 375 bpm. **(D)** Variance of the recovery beat cycle lengths for the entire digoxin data set (black), and Cluster 1(gray) and 2 (white). Cluster 2 variance was significantly smaller than Cluster 1 at 300 and 375 bpm.

We also investigated the average incidence of Cluster 2 beats following rapid pacing in order to determine which experimental conditions produced more OE beats. Figure 8A demonstrates that temperature (open and filled bars) did not significantly affect OE beat incidence per animal during digoxin or hypercalcemia (black and gray bars). Further, during hypothermia, digoxin produced significantly more OE beats than hypercalcemia.

### Mixed population effects

Figure 1B suggests that digoxin decreases RCL, but Figure 7A suggests that the population of Cluster 2 beats increases at faster pacing rates. Specifically Cluster 2 beats at 200 bpm account for 2 out of 42 beats, while at 375 bpm they account for 33 out of 66 beats. Thus, the decrease observed in Figure 1 may be a result of a switch at faster pacing rates from overdrive suppressed to overdrive excited beats. Figure 8B summarizes what happens to uncorrected RCL if Cluster 1 and 2 beats (gray and white respectively) are stratified from all recovery beats (black), and Figure 8C further demonstrates that the clustering algorithm accentuates the decrease in RCL ratio attributable to Cluster 2 beats. Furthermore, the RCL ratio demonstrates a similar relationship for stratified Cluster 2 beats, suggesting that the RCL ratio retains any relationship that RCL would have uncovered.

Stratifying between Cluster 1 and Cluster 2 beats also resulted in a much smaller variance for Cluster 2 beats for pacing rates greater than 250 bpm (Figure 8D), suggesting that OE beats occurred within a narrow time window. However, these Cluster 2 beats did not manifest any significant changes in variance as a result of pacing rate, suggesting that these beats occurred within a narrow time window regardless of pacing rate.

### Digoxin + tetrodotoxin

**Figure 9:**
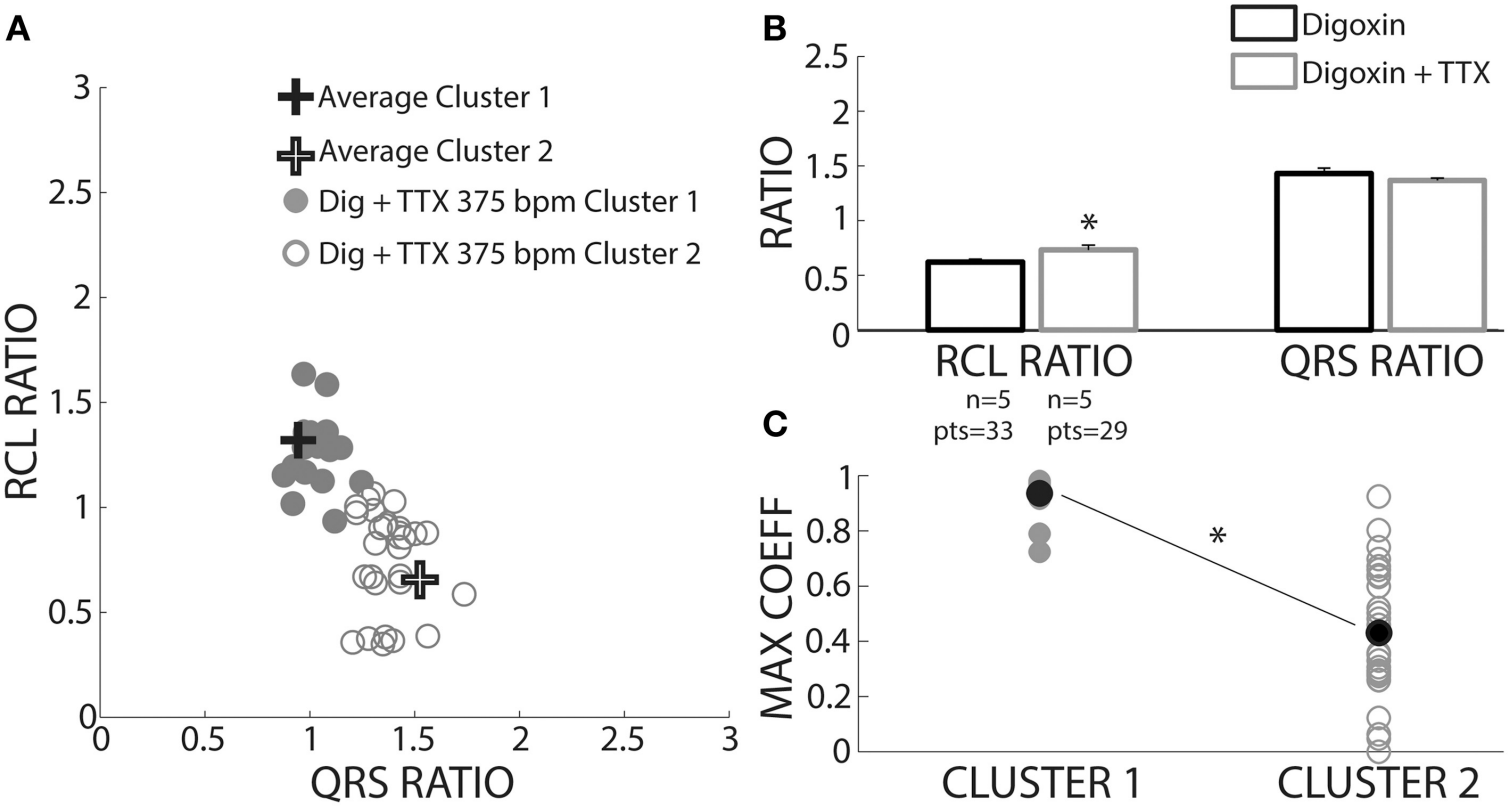
**TTX prolongs RCL without altering QRS of recovery beats. (A)** Centroids of Clusters 1 and 2 derived from Figure 4 are plotted as +. Each Digoxin + TTX beat was classified based on least-squares distance from the centroid in either Cluster 1 (gray filled circles) or 2 (gray open circles). **(B)** Cluster 2 data from digoxin and digoxin + TTX reveals only RCL ratio increased with TTX (*n* = 5, ^*^p < 0.05). **(C)** Cross-correlation analysis for QRS morphology reveals that Cluster 2 (*n* = 5 hearts, gray open circles) beat maximum correlation coefficients were significantly lower than Cluster 1 (*n* = 4 hearts, gray filled circles). Averages for each data set are shown as black filled circles.

We sought to prospectively test the performance of this algorithm by utilizing a pharmacologic protocol previously demonstrated to reduce calcium mediated arrhythmia vulnerability (Rosen and Danilo, 1980; Radwanski et al., 2013). Hearts were perfused with digoxin and tetrodotoxin (TTX) and assigned to clusters based on the shortest distance to *a priori* cluster centroids determined from Figure 4. Hearts were only paced at 375 bpm to preferentially elicit OE beats. As with digoxin, Cluster 2 beat (Figure 9A, open circles) RCL ratios were smaller (0.73 ± 0.04 vs. 1.25 ± 0.04) and QRS ratios larger (1.37 ± 0.02 vs. 1.02 ± 0.02) relative to Cluster 1 beats. Importantly, while TTX did not change the QRS ratio for Cluster 2 beats, it significantly increased the RCL ratio (Figure 9B, gray bars).

A cross-correlation analysis was also performed to determine QRS morphology similarity between recovery beats and pre-paced beats. The maximum cross-correlation coefficient from each analysis was determined for each recovery beat (Figure 9C). It was found that Cluster 2 beats had significantly smaller maximum correlation coefficients, indicating that QRS morphologies differed more from pre-paced beats. Stratified beat sensitivity and specificity were calculated using the maximum cross-correlation coefficient as the gold standard. Our clustering algorithm yielded high sensitivity (0.93) and specificity (0.94) relative to the cross-correlation algorithm (Table 1).

**Table 1:**
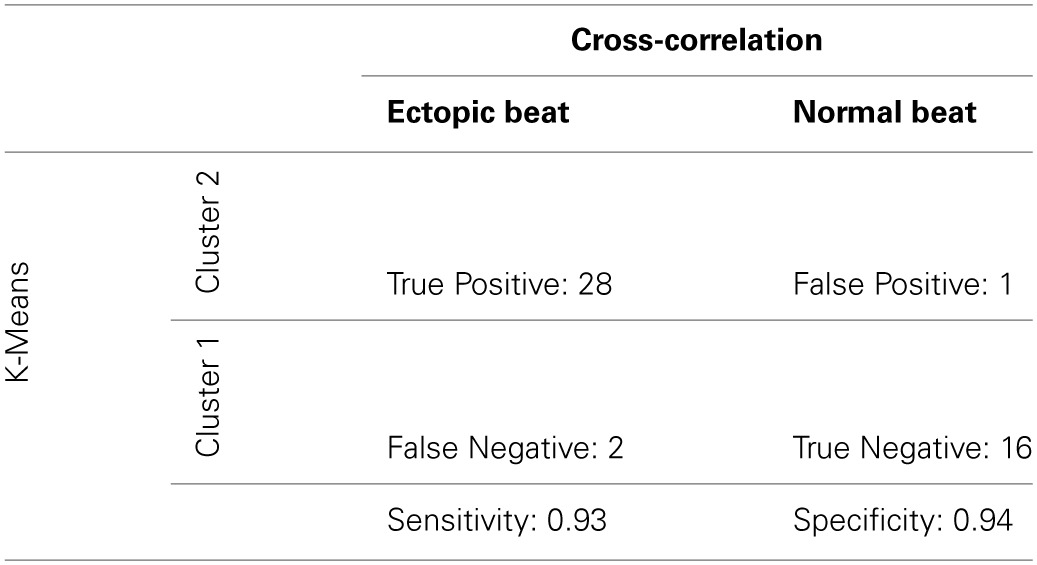
**Truth table: sensitivity and specificity of digoxin + TTX stratified beats**.

## Discussion

Rapid pacing is known to induce two types of recovery beats following the cessation of pacing: an interval of quiescence greater than or equal to the native rhythm, or a shortened interval. While we recapitulated previous studies demonstrating that RCL is proportional to pacing rate under control conditions (Malfatto et al., 1988; Iinuma et al., 1989) and inversely proportional during perfusion with a cardiac glycoside (Malfatto et al., 1988; Vos et al., 1990), the relationship observed for digoxin in Figure 1B may be misleading. More precisely, our beat classification system demonstrates that two distinct mechanistic populations compose recovery beats. The effect of grouping these mixed populations together would therefore significantly underestimate the magnitude of RCL shortening at very fast pacing rates. Therefore, in this study we present a methodology to differentiate between normal overdrive suppressed (OS) and arrhythmogenic excited (OE) rhythms independent of experimental intervention or inter-animal variability.

Previous methods for analyzing triggered activity (TA) in whole hearts have included analyzing recovery beats: (1) of ventricular origin, (2) that have a shorter cycle length than the longest post-paced R-R interval, (3) with preceding delayed afterdepolarizations (DADs), or (4) with preceding spontaneous calcium releases (Malfatto et al., 1988; Furukawa et al., 1990; Vos et al., 1990; Plummer et al., 2011). The use of raw RCL and QRS values resulted in preferentially clustering based on RCL values. Moreover, native rhythms and QRS complexes were altered by certain interventions, thereby affecting the ability to identify normal and arrhythmogenic beats based on raw RCL and QRS values. Therefore, we sought a simple and robust method for differentiating between recovery beats.

Our methodology utilized both normalizing the recovery beat cycle length to the pre-paced beat cycle length and the recovery beat QRS duration to the pre-paced beat QRS duration. The finding that OE beat RCL ratios reduced in response to increased pacing rate is consistent with previous reports of similar measurements using normalized recovery beat cycle length to post-pacing R-R interval as a mechanistic stratifier (Vos et al., 1990). Indeed, RCL and RCL ratio for OE beats were significantly smaller when compared to OS beats, consistent with OE beat behavior. Additionally, the QRS normalization data suggests that OE beats may occur lower in the ventricular conduction system or ventricular tissue than OS beats, as has been previously suggested (Burashnikov and Antzelevitch, 2000).

### Overdrive – suppression – normothermia

Our results demonstrated that control hearts only exhibited beats classified as OS, consistent with previous studies (Malfatto et al., 1988), and characteristic of normal automaticity. Other groups have proposed that OS may be caused by rapid pacing hyperpolarizing the membrane in response to activation of the sodium-potassium ATPase (NKA). (Vassalle, 1970; Abete and Vassalle, 1988) In contrast, the RCL ratio of OS beats during hypercalcemia was insensitive to pacing rate. To our knowledge, this is the first report of the effects of hypercalcemia on OS beats in whole heart preparations. Potential explanations for this finding may be that rapid pacing during hypercalcemia increases diastolic intracellular calcium more than under normocalcemic extracellular concentrations (Harding et al., 1993), thus augmenting NCX forward mode calcium extrusion, thereby increasing resting membrane potential (Hobai and O'Rourke, 2000), and perhaps making tissue hyperexcitable. Previous studies found that purkinje fibers recover more quickly from overdrive-induced hyperpolarization (Musso and Vassalle, 1982), or have an increased rate of diastolic depolarization (Temte and Davis, 1967), when placed in high calcium solutions.

Directly inhibiting NKA with digoxin created OS beats insensitive to pacing rates. This is consistent with previous research that demonstrated cardiac glycosides can blunt the prolongation of the recovery interval typically seen under control conditions (Hogan et al., 1973). Furthermore, others have demonstrated that the glycoside ouabain abolishes overdrive hyperpolarization and shifts the maximum diastolic potential to more positive values in feline purkinje fibers (Browning et al., 1979), guinea pig and sheep ventricular muscle, and purkinje fibers (Cohen et al., 1982). In summary, the increase in RCL could be due to membrane hyperpolarization by NKA and, subsequently, NKA inhibition could blunt hyperpolarization and pacing rate-RCL dependence.

### Overdrive – suppression – hypothermia

The expected relationship between OS RCL and pacing rate is maintained for control conditions, but surprisingly increasing pacing rate during hypothermic hypercalcemia decreased OS RCL ratio. Sensitivity and specificity for the ratio analysis was high (0.8 and 0.98, respectively) using a QRS cross-correlation analysis as the gold standard (Kyle et al., 2009). Furthermore, cross-correlation analysis revealed the same RCL to pacing rate relationship for OS beats. Both analyses suggest the surprising finding that rapid pacing may actually decrease the RCL of hypothermia + hypercalcemia OS recovery beats. As discussed above during normothermia, hypercalcemia may increase NCX activity and depolarize the membrane. Previous studies under severe hypothermia (21–22°C) have suggested that NCX plays a larger role in calcium sequestration (Puglisi et al., 1996), which may augment membrane potential depolarization during rapid pacing, perhaps resulting in automatic recovery beats that return earlier. This hypothesis requires validation, however.

Lastly, OS RCL during hypothermic digoxin was insensitive to pacing rate. Unlike hypercalcemia which directly increases intracellular calcium by increasing the calcium driving force, NKA inhibition increases intracellular sodium prior to calcium. Therefore, NCX may not significantly depolarize the membrane during hypothermic digoxin and rapid pacing because intracellular sodium and calcium rise concurrently.

### Overdrive – excitation – normothermia

Once again, control conditions never produced an OE beat in this study. In six hearts perfused with hypercalcemia, only two hearts produced OE beats and, therefore, conclusions cannot be readily drawn from this protocol. For digoxin, relatively slow pacing rates did not generate OE beats in sufficient hearts for statistical comparison or discussion.

Yet, RCL variance for digoxin OE beats was significantly smaller than for OS beats, suggesting that the mechanisms underlying OE beats may be more temporally deterministic than mechanisms underlying OS beats. Interestingly, RCL variance for OE beats did not change with pacing rate, which may be inconsistent with a previous study that demonstrated that increasing pacing rate in intact tissue decreases the standard deviation of spontaneous calcium release timing (Wasserstrom et al., 2010). Furthermore, post-hoc analysis of another study suggests that spontaneous calcium release timing variability in whole hearts decreases as a function of pacing rate as well (Plummer et al., 2011). This intriguing discrepancy could be a result of differences in spontaneous calcium releases and their subsequent fully propagated TA response.

### Overdrive – excitation – hypothermia

In contrast to normothermia, all five hearts perfused with hypothermic hypercalcemic solutions exhibited OE beats at the fastest pacing rates, but not at the slowest pacing rate, thus precluding a discussion of mechanisms. However, during hypothermic digoxin, the OE RCL ratio decreased as pacing rate increased, consistent with previous studies demonstrating that rapid pacing decreases RCL of TA (Furukawa et al., 1990; Plummer et al., 2011). The proposed mechanism of OE is due to a rise in diastolic intracellular calcium levels leading to increased probability of a spontaneous calcium release from the sarcoplasmic reticulum causing forward mode calcium extrusion through NCX thereby depolarizing the membrane prematurely (Wier and Beuckelmann, 1989; Schlotthauer and Bers, 2000). As mentioned previously, hypothermia can raise resting membrane potential and make tissue more likely to exhibit a triggered beat. In short, hypothermic digoxin not only produced the most OE beats, but the behavior of these beats was consistent with TA.

### Sodium channel inhibition and excited beats

We also investigated whether inhibition of TTX-sensitive sodium channels could impact the timing of OE beats. OE beat RCL ratio was prolonged in the presence of the sodium channel inhibitor tetrodotoxin (TTX). This is consistent with the finding that non-specific sodium channel blockers can delay the time to spontaneous calcium release (Lee et al., 2012). Indeed, previous studies have suggested that sodium channel inhibitors can act as antiarrhythmic agents, decreasing DAD amplitude and preventing OE beats (Rosen and Danilo, 1980; Radwanski et al., 2013). A few mechanisms have been proposed to explain the delay of onset of OE beats, such as decreased membrane excitability secondary to sodium channel inhibition. However, the native QRS duration and QRS ratio of OE beats remained unaffected by this degree of sodium channel inhibition, suggesting that membrane excitability was not altered. Further, we have shown that this concentration of TTX did not modify membrane excitability in an Andersen-Tawil syndrome guinea pig model of calcium mediated arrhythmias (Radwanski et al., 2013). Another possible mechanism is that TTX prevents intracellular calcium accumulation (Torres et al., 2010). While further investigation is needed to understand the mechanisms by which sodium channel inhibition affects OE beats, these results demonstrate that inhibiting sodium channels prolongs the time to the OE.

### Algorithmic performance

The maximum coefficient from cross-correlation analysis of QRS complexes has previously been used to identify premature ectopic beats of ventricular origin, and resulted in high specificity and sensitivity values indicating that it is a good predictor for identifying ectopic beats (Kyle et al., 2009). Therefore, in this study, the lower maximum correlation coefficient found for OE beats suggests that our prospective analysis can distinguish between OS and OE beats. Furthermore, the sensitivity and specificity of our analysis validated against the maximum coefficient was high.

## Conclusions

We present a new methodology for distinguishing between automatic and triggered activity. OS beats exhibited automatic behavior supported by data demonstrating that increasing pacing rate increased RCL ratio in control conditions. During hypothermic digoxin conditions OE beats manifested triggered activity characteristics as RCL ratio decreased. Moreover, OE beats manifested overdrive acceleration. These OE beats also exhibited larger QRS ratios and smaller RCL ratios when compared to OS beats within experimental conditions. Hypercalcemia and digoxin were able to create OE beats, whereas control conditions could not, supportive of the hypothesis that calcium overload underlies TA. Hypothermic digoxin produced the most OE beats. Sodium channel inhibition, which has been shown to ameliorate calcium mediated arrhythmia burden, increased the time to initiation of an OE beat. The prospective analysis performed well, suggesting that our methodology is able to distinguish between OS and OE beats. Our quantitative methodology presents a relatively simple technique for differentiating between automatic and triggered activity.

## Limitations

The electromechanical uncoupler 2,3-diacetylmonoxime (BDM or DAM) was used in this study. This compound alters intracellular calcium concentrations and handling, and therefore may impact OE beat formation (Backx et al., 1994). Therefore, it may impact the occurrence of OE beats. Nevertheless, all experimental conditions were exposed to 2,3-diacetylmonoxime and thus its impact would have been included for all beats.

## Disclosures

The authors have submitted a University Invention Disclosure to Virginia Tech in regards to the quantitative methodology described in this study.

### Conflict of interest statement

The authors declare that the research was conducted in the absence of any commercial or financial relationships that could be construed as a potential conflict of interest.

## Abbreviations

BCL: Basic Cycle Length
DAD: Delayed Afterdepolarization
OE: Overdrive Excitation
OS: Overdrive Suppression
NCX: Sodium-Calcium Exchanger
NKA: Sodium-Potassium ATPase
TTX: Tetrodotoxin
RCL: Recovery Beat Cycle Length
TA: Triggered Activity.
